# Terahertz Broadband Absorber Based on a Combined Circular Disc Structure

**DOI:** 10.3390/mi12111290

**Published:** 2021-10-21

**Authors:** Meihong Huang, Kaihua Wei, Pinghui Wu, Danyang Xu, Yan Xu

**Affiliations:** 1 College of Transportation and Navigation, Quanzhou Normal University, Quanzhou 362000, China; plum_huang@qztc.edu.cn; 2 School of Automation, Hangzhou Dianzi University, Hangzhou 310018, China; weikaihua@hdu.edu.cn; 3 Fujian Provincial Key Laboratory for Advanced Micro-Nano Photonics Technology and Devices, Quanzhou Normal University, Quanzhou 362000, China; phwu@zju.edu.cn; 4 College of Science, Zhejiang University of Technology, Hangzhou 310023, China; xudanyang@zjut.edu.cn; 5 School of Science, Huzhou University, Huzhou 313000, China

**Keywords:** terahertz, perfect absorption, broadband, ring-disk structure, polarization insensitive

## Abstract

To solve the problem of complex structure and narrow absorption band of most of today′s terahertz absorbers, this paper proposes and utilizes the finite element (COMSOL) method to numerically simulate a broadband absorber based on a straightforward periodic structure consisting of a disk and concentric ring. The final results show that our designed absorber has an absorption rate of over 99% in the broadband range of 9.06 THz to 9.8 THz and an average of over 97.7% in the ultra-broadband range of 8.62 THz to 10 THz. The reason for the high absorption is explained by the depiction of the electric field on the absorber surface at different frequencies. In addition, the materials for the top pattern of the absorber are replaced by Cu, Ag, or Al, and the absorber still achieves perfect absorption with different metal materials. Due to the perfect symmetry of the absorber structure, the absorber is very polarization-insensitive. The overall design is simple, easy to process and production. Therefore, our research will offer great potential for applications in areas such as terahertz electromagnetic stealth, sensing, and thermal imaging.

## 1. Introduction

THz technology has received increasing attention and interest these years [1]. Terahertz waves are high-frequency electromagnetic waves in the frequency band of 0.1 THz to 10 THz, which occupy a critical position in the electromagnetic wave spectrum [2]. As a transition interval between electronics and optics, the terahertz band is widely used in communication, detection, sensing, stealth, and other fields because of its many unique advantages such as low photon energy, short pulse, and frequency [3,4,5,6,7,8,9]. The terahertz absorber based on electromagnetic metamaterials is one of the important devices applied in electromagnetic detection and stealth. Besides, the perfect absorber of metamaterials in the terahertz band has been a research hotspot and challenge in recent years. The desired electromagnetic characteristic parameters can be obtained by changing the composition structure, geometric parameters, and arrangement of metamaterials [10,11,12], which provides us with an effective approach to the design and application of terahertz perfect absorbers. For example, an absorber composed of the structure of a metal ring, a silica spacer, and a vanadium dioxide film was proposed by Lingling Chen et al., which enables single-band absorption in the terahertz band [13]. The absorber has a very simple structure consisting of only circular rings. In 2020, Wangyang Li et al. proposed a tunable dual-band terahertz perfect metamaterial absorber (MMA), composed of two stacked square STO resonator structures and a metal substrate [14]. The repeated double-ring structure is responsible for the formation of the dual-band absorption and provides the basis and idea for the design of multi-band, broadband absorbers. Moreover, in 2020, Yuqian Wang et al. designed a Dirac semimetal-based absorber that consists of a square-wave oscillator with four BDS films and a closed-loop to achieve multiband absorption in the terahertz band [15]. However, broadband absorption is a greater hotspot and difficulty in scientific research for the perfect absorber of metamaterials. Broadband absorption can better meet our practical needs than most single-band, dual-band, and multi-band absorbers [16,17,18,19,20].

In practice, there are two general methods to achieve broadband absorbers. The first is through the planar construction method [21,22,23], the combination and arrangement of different patterns to change the electromagnetic properties of the absorber. The second method is multi-layer addition [24,25,26]. This method strengthens the interaction between layers by increasing the number of layers of absorption layer, to achieve the purpose of changing the characteristics of absorption layer. In addition, we study the resonance mechanism of the two methods. The resonance mechanism of both methods is to achieve high broadband absorption through the superposition of resonances of different frequencies. Although the multilayer stacking method can achieve broadband absorption, the design of multilayer structure is usually very difficult in the actual process of surface preparation [27,28,29]. Because, in actual production, precise alignment between the layer and the size of the absorber we design is required, usually at the micron and nanometer level. This means that each stack of the absorber structure makes the process more geometrically difficult. Therefore, it is of great significance to design a kind of traditional metamaterial which can realize broadband absorption.

In this study, we designed a metamaterial which is based on a metal-dielectric-metal structure [30,31,32,33,34] for a broadband perfect absorber in the terahertz band. The top pattern consists of a closed gold ring and a disc, which provides excellent performance and simple construction. The middle layer is made of silicon dioxide as a dielectric layer and the bottom layer is made of gold film as a reflective layer. Through simulations, we have studied the effect of different structural parameters, polarization angles and incidence angles on the absorption effect of the performance and used this as a basis for optimizing the structural parameters. We analyzed the absorption mechanism and polarization characteristics by combining the distribution of electric field and current density on the surface of metamaterials. Finally, we obtained the planar combination structure of circle and disk as an effective method to design broadband absorbers. Finally, the results show that our designed absorber has a polarization-insensitive absorption rate of over 99% in the broadband range of 9.06 THz to 9.8 THz and over 97.7% in the ultra-broadband range of 8.62 THz to 10 THz. This suggests that our research would have enormous potential for applications in terahertz electromagnetic stealth, sensing, and thermal imaging.

## 2. Mathematical and Experimental Methods

**Figure 1:**
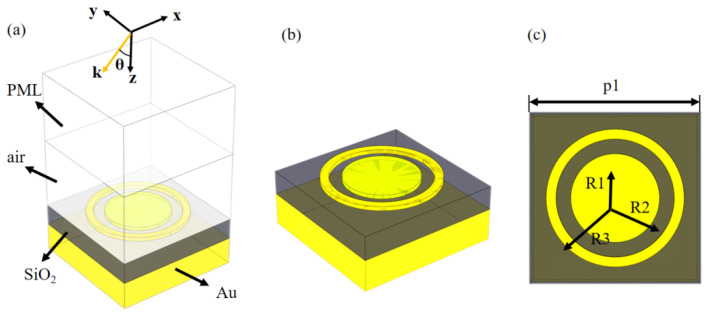
(**a**) Front view of absorber, (**b**) periodic unit of absorber, (**c**) top view of absorber.

The structure proposed in this paper is shown in Figure 1b, and the absorber consists of three units, a periodic structure composed of metal-dielectric-metal. We chose gold as the target metal because of its chemical stability to ensure a higher environmental suitability of the absorber. In the design structure of the absorber, the first layer from the bottom up is the reflective layer, which ensures that there are no transmitted electromagnetic waves. The second and third layers are the dielectric and absorption layers respectively, which ensure the loss of electromagnetic waves. The bottom layer of the absorber is a continuous gold film with a thickness h = 8 µm and the dielectric layer with a thickness d of 6 µm silica with a relative dielectric constant $\varepsilon_{p}\;$= 1.46. The gold pattern in the top layer of the absorber is a combination of rings and disks with a thickness t of 0.1 µm. We used the finite element method in COMSOL simulation platform to simulate and optimize the metamaterial absorber with the following optimal parameters: p1 = 35 μm, R1 = 7 μm, R2 = 12 μm, R3 = 14 μm.

We implement the periodic array with a single structural unit by setting Floquet periodic boundary conditions. When the electromagnetic wave is incident perpendicular to the absorber surface, the electric and magnetic fields are parallel to the x and y axis directions respectively. The z-direction is set to perfectly match the layer, as shown in Figure 1a. In the simulation, all the metallic layers are composed of gold material. In the closed-loop disc structure and geometrical parameters we designed, the electromagnetic resonance of a specific frequency occurs when the electromagnetic wave interacts with the absorber. By means of the Drude model [35,36,37]$\;\varepsilon =1-\frac{\omega_{p}^{2}}{\omega \left(\mathrm{iwt}\right)}$. We can calculate the dielectric constant of gold, where the volume plasma frequency is $\omega_{p}=1.37^{16}s^{-1}$ and collision frequency $\omega_{t}=1.23^{14}s^{-1}$ [38]. When the thickness of the underlying metal is greater than the maximum skin depth of the metal in terahertz, spectrum the transmittance of the absorber T = 0. Therefore, the absorptance of the absorber can be simplified by A(ω) = 1 − R(ω) − T(ω) [39,40,41,42,43] as A(ω) = 1 − R(ω), where R is the reflectance of the absorber. In this paper, the thickness of the reflective gold film is 8 μm, which is bigger than the skinning depth and ensures that the transmittance T(ω) = 0.

Because the design of the periodic metal structure array on top of the absorber affects the impedance of the absorber, we can adjust the structural parameters of the absorber, and material parameters to match the impedance of the absorber with the free space [44,45,46], so that the reflection coefficient tends to zero and the absorber absorption rate tends to 1. The dielectric layer of the absorber provides enough space for the propagation of electromagnetic waves in the absorber and realizes the loss of electromagnetic waves in the absorber. The absorber is insensitive to polarization, owing to the perfect symmetry of our closed-loop disc combination structure. Our closed-loop disc structure will have a higher impedance than the short cut open-loop form of the metal pattern. It means that the absorber is more easily impedance matched to space to achieve high absorption rates over a wide frequency band and small feature size.

## 3. Results and Discussion

**Figure 2:**
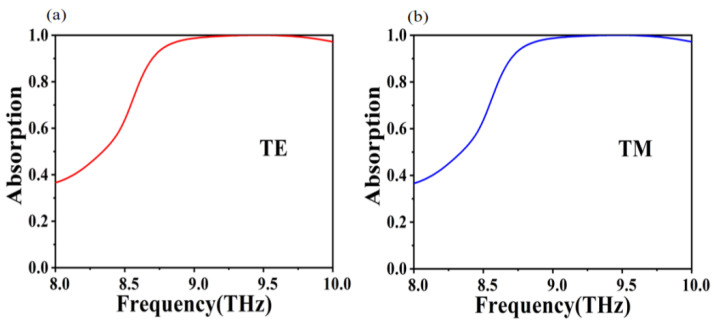
The absorption simulation curve of the absorber at normal incidence in: (**a**) TE, (**b**) TM.

In order to construct an absorber with a broadband perfect absorption effect and a simple structure, we have designed a perfect absorber based on a closed-loop disc combination structure. Through the COMSOL simulation platform, as shown in the Figure 2, we calculated the absorptance of the absorber in TE and TM modes at positive incidence, respectively. As can be seen from the graph, our designed absorber is not only insensitive to polarization, but also has an absorption >99% over the broadband range of 9.06 to 9.8 THz and still achieves a high average absorption of >97.7% over the ultra-wideband range of 8.62 to 10 THz. The reason for the polarization insensitivity is that the nanostructure unit of the absorber shows perfect symmetry [44,45,46,47]. Our absorber has more advantages compared to the others, as shown in Table 1 [47,48,49,50]. Compared to the 5-layer structure and even the complex structure with more than 10 layers in Table 1, our absorber with only 3 layers is very simple and easy to fabricate. Moreover, it has a large broad absorption bandwidth. The absorption bandwidth is 1.38 THz, which is a significant improvement compared to the absorbers in Table 1.

**Figure 3:**
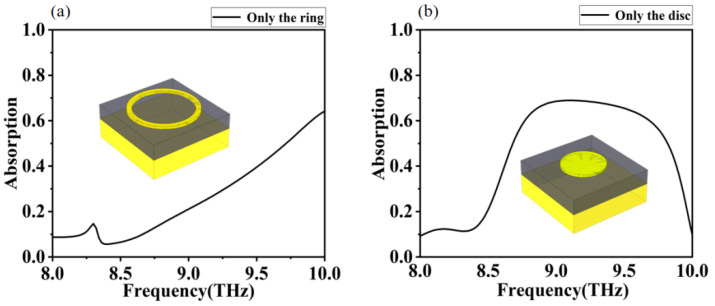
Absorption simulation curves of the absorber with different structures: (**a**) only the ring, (**b**) only the disc.

**Figure 4:**
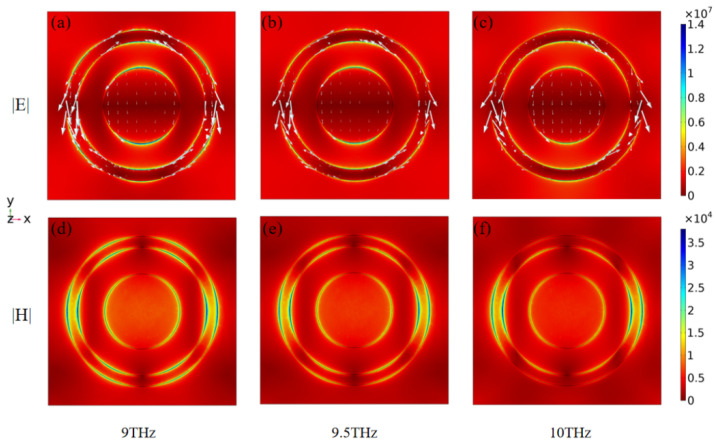
Current density distribution simulation diagram of electromagnetic field of absorber at different frequencies. (**a**) TE mode, f = 9 THz, (**b**) TE mode, f = 9.5 THz, (**c**) TE mode, f = 10 THz, (**d**) TM mode, f = 9 THz, (**e**) TM mode, f = 9.5 THz, (**f**) TM mode, f = 10 THz (The size and direction of the white arrows indicate the direction of current density respectively).

In order to gain a deeper understanding of the principle of the absorber producing optimum absorption, we have plotted the absorption rate of the absorber with only the closed circle and only the disc as shown in Figure 3. The distribution of the electromagnetic field at the top of the absorber for different modes (TE, TM), at different frequencies, and the distribution of the current density are shown in Figure 4.

In Figure 3, we calculate the absorptivity of an absorber with only a closed ring and only a disk. It can be noticed that both structures do not have a high absorption rate (less than 70%) in the 8.0–10.0 THz range, as shown in Figure 3a,b. This is because the absorber under only the ring or disc structure does not form an effective resonant coupling with the external terahertz wave. However, the absorbers in the first structure have an enhanced absorption effect as the frequency increases. The absorbers in the second structure show a significant increase in absorption in the 8.7–9.7 THz range compared to the other bands. Both structures have some resonance potential at high frequencies, which provides ideas for our design. So, we propose an innovative closed-loop disk combination structure. The structure increases the interaction between ring and disk and achieves high absorption performance of the absorber. In Figure 4, we depict the electric field and current density distributions on the surface of the closed-loop disc for electromagnetic waves in TE, TM mode at the frequencies of 9 THz, 9.5 THz, and 10 THz, respectively. To explain why the absorber produces a perfect absorption, we plotted the distribution of electric field and current density corresponding to these three frequencies together with the absorption curve. It is easy to understand that the high absorption rate of the absorber over a wide frequency band is mainly caused by the resonant coupling of the combined closed ring and disc structure [51,52]. By comparing the electric field distribution at 9 THz as well as at 10 THz, we can see that resonant coupling performs a significant function in absorbing low and high frequency bands. Since the polarization directions in TE and TM modes are different, it can be found from Figure 4 that the position of resonance is determined by the polarization direction. At TE mode, the resonant coupling of the combined closed-loop and disc structure in the longitudinal direction is relatively stable and prominent, compared to the resonant coupling of the combined closed-loop and disc structure in the transverse direction in TM mode. The electric field distribution on the closed-loop disc shows that the terahertz wave induces an induced strong electromagnetic field on the metal ring and disc. The closed-loop disc and the underlying metal are separated by a dielectric layer, which can be equated to an electric dipole. As the electromagnetic waves act on the closed-loop disc, the charge builds up and the electric dipole strengthens. The strong electric dipole resonates strongly with the external electromagnetic wave on the disc and ring, culminating in a high absorption [53,54].

**Figure 5:**
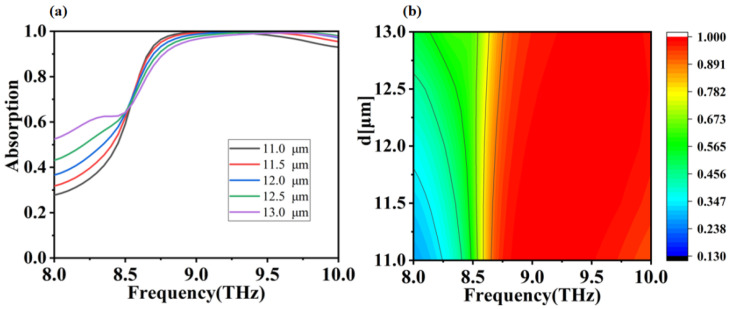
(**a**,**b**) are absorption simulation images of scanning medium layer thickness (11–13 μm) at frequency from 8.0 to 10.0 THz, respectively.

**Figure 6:**
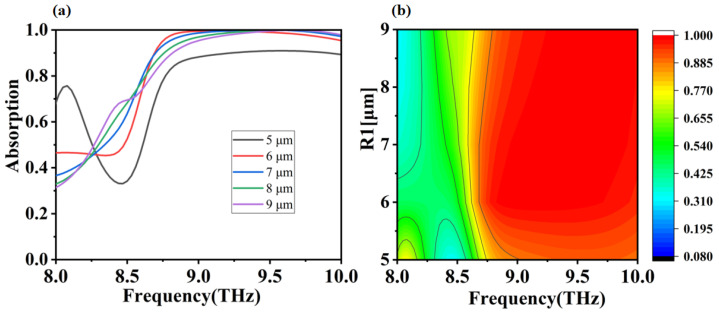
(**a**,**b**) are absorption simulation images of different disk radii R1 (5–9 μm) scanned at frequencies from 8.0 to 10.0 THz, respectively.

In the above discussion, we have calculated and analyzed the absorption performance of a closed-loop and disc combination absorber under ideal conditions. Let us now consider one of the main factors affecting the absorption performance of the absorber in practice, which is the process error in the process production. In actual process production, the precise alignment of the absorber layers and the accurate control of the absorber structure parameters are two major difficulties. Owing to the very simple structure of the absorber we have designed, this is much less difficult in practice, but there are still errors in the process that need to be considered. The radius of the disc and the height of the dielectric layer largely influence the electromagnetic resonance of the absorber and thus determine the absorption effect. In the following we will analyze and discuss the influence of these two parameters on the performance of the absorber. In Figure 5 and Figure 6 we depict the absorption curves of absorbers with different dielectric layer thicknesses and different disc radii for positive incidence electric field. From Figure 5, we can see that there is a significant redshift in the absorption curve as the thickness of the dielectric layer changes, but in general the absorber is still able to maintain a high level of absorption within a broadband, which implies a good tolerance to production errors within a small range of dielectric layer thickness in-process production. We can also see from Figure 5 that as the dielectric layer thickness increases, the absorption decreases at low frequencies and increases at high frequencies. This is caused by the SPP of metallic materials in the terahertz band, but losses reduce the surface plasma mass, thus making the absorption effect less effective due to conductivity, polarization, hysteresis, and a certain propagation distance [55,56,57]. In fact, the dielectric layer provides the transmission space and the consumption path for the terahertz waves entering the absorber and plays a decisive role in the performance of the absorber. In terms of impedance matching theory, this means that variations in the thickness of the dielectric layer affect the impedance matching between the absorber and the free space, and ultimately the absorber′s absorption effectiveness [58,59]. From Figure 6 we can see that the absorption curve of the absorber is different from the radius of the disk. With the change of disk radius, the absorption curve has the phenomenon of red shift. However, the disk radius is 5 μm, and the absorption effect is much lower than other parameters. This is because when the disk radius is 5 μm, the disk-ring interaction is weak and the entire magnetic dipole in the absorber cannot be excited effectively, resulting in poor absorption. When the disc radius varies from 6 μm to 9 μm, the absorber effect is still strong in the broadband, which also means that the absorber has a good process tolerance for process production.

**Figure 7:**
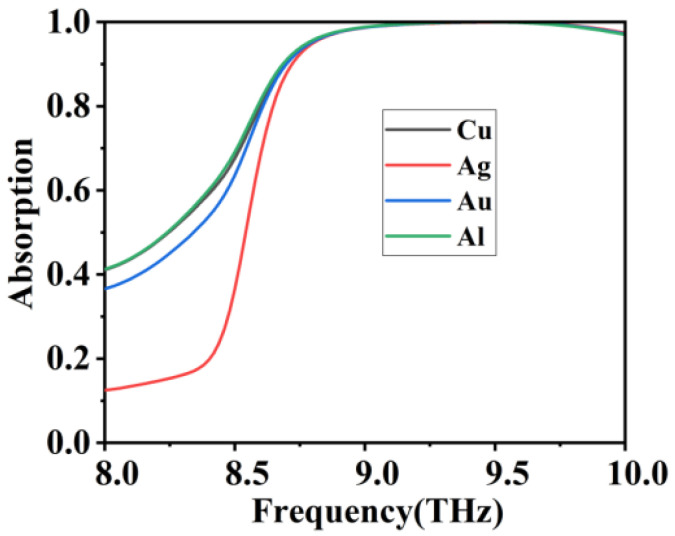
Absorption simulation curves of different materials.

Moreover, we calculated the absorption behavior with various material composition. The type of material used to form the ring and disk at the top of the absorber is changed without changing the structural parameters of the designed absorber. The effects of different materials on the absorption properties were observed. Here we use Cu and Ag in the same group as Au and Al with low refractive index. From Figure 7 we can see that the absorber with the combination of closed-loop discs of different materials still maintains a high absorption effect at high frequencies and is almost identical, with only some differences at lower frequencies. This is because Au, Ag, and Al are all low refractive index metals and Cu, Ag, and Au belong to the same main group of elements with similar properties [60,61,62,63]. They all give rise to SPP in the terahertz band. The results show the adaptability of our closed-loop disc absorbers to a wide range of materials. Moreover, the designed absorber has an excellent absorption effect, a simple structure that is easy to fabricate and a good adaptability to materials.

## 4. Conclusions

Overall, we have designed a metamaterial wideband terahertz ideal absorber with simple structure. By describing the distribution of electric field intensity and current density on the surface of the absorber, the mechanism of the absorber producing high absorbent was studied. Finally, the results show the absorption rates of the absorber exceed 99% in the broadband range from 9.06 THz to 9.8 THz and average over 97.7% in the ultra-broadband range from 8.62 THz to 10 THz. In addition, the materials for the top pattern of the absorber are replaced by Cu, Ag, and Al, and the absorber still achieves perfect absorption with different metal materials. This means that our research will have great potential for applications in areas such as terahertz electromagnetic stealth, sensing, and thermal imaging. Compared to the absorbers proposed in recent years for the terahertz band, our absorber has a lower process difficulty, higher absorption rate and bandwidth, and better universality, providing a new idea for the study of metamaterial perfect absorbers for the terahertz band.

## Figures and Tables

**Table 1:** Some broadband absorbers in the terahertz band in recent years.

References	Absorption Bandwidth	Number of Absorber Layers	Processing Difficulty	Absorption Rate
[44]	1.25~2.13 THz	5	High	More than 95%
[45]	1.05~2.35 THz	8	High	More than 95%
[46]	1.2~2 THz	greater than 10	High	More than 95%
[47]	1.23~1.68 THz	3	Low	More than 95%
proposed	8.62~10 THz	3	Low	More than 95%
